# Elucidating the Synergistic Effect of the PrimeC Combination for Amyotrophic Lateral Sclerosis in Human Induced Pluripotent Stem Cell-Derived Motor Neurons and Mouse Models

**DOI:** 10.3390/ph18040524

**Published:** 2025-04-03

**Authors:** Shiran Salomon-Zimri, Nitai Kerem, Gabriel R. Linares, Niva Russek-Blum, Justin K. Ichida, Ferenc Tracik

**Affiliations:** 1 NeuroSense Therapeutics, Ltd., Ha-Menofim 11, Herzliya 4672562, Israel; nitai@neurosense-tx.com (N.K.); niva@neurosense-tx.com (N.R.-B.); ferenc@neurosense-tx.com (F.T.); 2 Department of Stem Cell Biology and Regenerative Medicine, Keck School of Medicine, University of Southern California, Los Angeles, CA 90033, USA; dr.gabriel.linares@gmail.com (G.R.L.); ichida@usc.edu (J.K.I.)

**Keywords:** amyotrophic lateral sclerosis, combination therapy, iPSCs, in vivo, in vitro, preclinical, synergism, mechanism of action

## Abstract

**Background:** Amyotrophic lateral sclerosis (ALS) is a multifactorial neurodegenerative disease characterized by the involvement of multiple pathways and mechanisms. The complexity of its pathophysiology is reflected in the diverse hypotheses relating to its underlying causes. Given this intricate interplay of processes, a combination therapy approach offers a promising strategy. Combination therapies have demonstrated significant success in treating complex diseases, where they aim to achieve synergistic therapeutic effects and reduce drug dosage. PrimeC is an oral combination treatment composed of a patented novel formulation consisting of specific and unique doses of two well-characterized drugs (ciprofloxacin and celecoxib). It aims to synergistically inhibit the progression of ALS by addressing key elements of its pathophysiology. **Objectives:** Demonstrating the synergistic effect of the PrimeC combination compared to each of its individual components, celecoxib and ciprofloxacin, and assessing its ability to improve the drug concentration profile and efficacy. **Methods:** The efficacy of the PrimeC combination was assessed in a survival assay using human induced pluripotent stem cell (iPSC)-derived motor neurons. Additionally, a drug profiling study was conducted, measuring drug levels in the brain and serum of C57BL mice treated with a single compound versus the combination. **Results:** Motor neurons modeling ALS treated with the PrimeC combination exhibited better survival rates compared to treatment with either individual compound alone. The enhanced efficacy of the combination was further supported by a drug concentration profiling study in rodents, demonstrating that the PrimeC combination resulted in increased ciprofloxacin concentrations in both brain tissue and serum—highlighting the optimized interaction and synergistic potential of its two comprising agents. **Conclusions:** Our findings support the potential of combination therapy as an effective strategy for ALS treatment. Specifically, the PrimeC combination demonstrated promising therapeutic effects, providing a strong rationale for its ongoing development as a targeted treatment for ALS.

## 1. Introduction

Amyotrophic lateral sclerosis (ALS) is a severe neurodegenerative disorder characterized by the progressive deterioration of motor neurons (MNs), resulting in muscle paralysis and eventual death within 2–5 years from onset [1]. The exact pathogenesis of ALS is not fully understood and involves various pathophysiological pathways, including neuroinflammation [2], iron accumulation [3], and microRNA (miRNA) dysregulation [4], among others. The multifaceted nature of ALS and the absence of a clearly defined mechanism suggest that a combination of pathways contributes to neurodegeneration in ALS, necessitating a multi-targeted therapeutic approach.

PrimeC is a novel proprietary formulation composed of unique doses of ciprofloxacin and celecoxib, aiming to synergistically inhibit the progression of ALS. These two compounds complement each other in targeting key elements of ALS pathophysiology, with each individual compound addressing distinct mechanisms, namely miRNA dysregulation, neuroinflammation, and iron accumulation. The synchronized release of PrimeC was designed to optimize pharmacokinetics and maximize synergistic effects.

Ciprofloxacin, a fluoroquinolone antibiotic, has been shown to modulate miRNA levels through mechanisms similar to other fluoroquinolones known to promote the association of RNA-binding proteins, such as TRBP, with the RISC-loading complex [5]. Imbalances in these processes are observed in ALS and other neurodegenerative diseases [6]. Ciprofloxacin also plays a role in the inhibition of NF-ƙB, thereby downregulating pro-inflammatory cytokines, leading to inhibition of neuroinflammation and resulting in the regulation of hepcidin levels, inhibiting further iron accumulation [7]. As such, ciprofloxacin functions as an iron chelator [8], with various examples of iron chelators reducing brain iron stores and exhibiting neuroprotective effects in neurodegenerative diseases [9].

Celecoxib, a non-steroidal anti-inflammatory drug (NSAID), is known for its selective COX-2 inhibition, complemented by COX-2-independent mechanisms contributing to its anti-inflammatory action [10]. The inhibition of COX-2 has been shown to affect key ALS pathologies, including glutamate-induced excitotoxicity [11], inflammation [12], ferroptosis [13], and oxidative stress [14], among others. Although celecoxib at a high dose has not been shown to be beneficial in a previous clinical study exploring the potential of treating ALS with celecoxib alone [15], low doses of celecoxib were shown to be effective in pain [16] and played a synergistic role with ciprofloxacin in regulating oxidative stress and inflammation in vivo [17].

Preclinical studies combining celecoxib with ciprofloxacin showed a synergistic effect in zebrafish ALS models, with benefits to motor function as well as to central nervous system (CNS) cell morphology [18].

In a Phase IIa clinical study aimed to assess PrimeC’s potential beneficial effects, results showed safety and tolerability in people living with ALS (PALS) [19]. This study also demonstrated, via relevant biomarkers (e.g., TDP-43, PgJ2, and LC3), a promising biological effect for the combination on mitigating disease progression. A subsequent Phase IIb clinical trial aiming to evaluate the safety, tolerability, and efficacy of PrimeC in PALS through a randomized, placebo-controlled, double-blind study (PARADIGM; NCT05357950), demonstrated consistently positive effects across all clinical outcomes (ALSFRS-R total score, sub-domains, SVC, etc.), with a favorable safety and tolerability profile. PrimeC treatment slowed disease progression, trended towards improved survival, demonstrated beneficial regulation of miRNA, and effectively modulated iron metabolism, as observed by regulation of transferrin and ferritin (unpublished data).

Combination therapy is most widely used in treating the most dreadful diseases, such as cancer, Parkinson’s disease, epilepsy, and acquired immunodeficiency syndrome (AIDS), and is gaining widespread recognition by scientists and clinicians as a novel therapeutic approach. By combining two well-characterized drugs in a unique formulation, we aim to achieve synergistic therapeutic effects while reducing dose requirements and minimizing toxicity.

To evaluate the synergistic effect of the PrimeC combination, two preclinical studies were conducted: (1) an in vitro study aimed to assess the synergistic effect of the PrimeC combination compared to each individual compound (ciprofloxacin and celecoxib) using human induced pluripotent stem cell (iPSC)-derived MNs carrying ALS-associated C9orf72 pathology; and (2) drug concentration profiling in C57BL mice.

These findings, together with the proposed mechanistic framework and supporting preclinical evidence, highlight the potential of PrimeC as a novel combination therapy for neurodegenerative diseases, particularly ALS. This integrated approach sets the stage for advancing PrimeC as an innovative therapeutic strategy in this field.

## 2. Results

### 2.1. PrimeC Combination Improves Survival of ALS Patient-Derived iMNs

**Figure 1:**
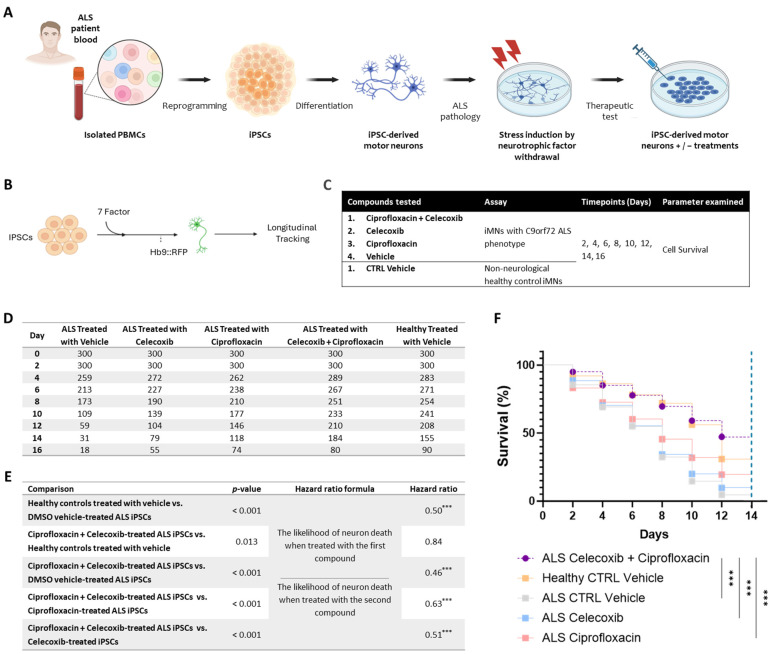
(**A**) Schematic overview of donor-specific iPSC generation. Peripheral blood samples were obtained from PALS (C9orf72 pathology) via the NINDS repository. (**B**) Schematic representation of the differentiation process of iPSCs into iMNs, followed by longitudinal survival tracking. (**C**) Study Design. ALS patient-derived iPSCs were treated with one of the indicated compounds or with DMSO vehicle control. Neuronal survival (cell count) was measured over a 16-day period, with survival assessed at the indicated time points. (**D**) Quantification of surviving iMNs across treatment conditions at each measured time point. All conditions started with 300 tracked iMNs on day 0, with cell survival assessed every other day. (**E**) Hazard ratio analysis comparing overall iMN survival between treatment conditions. (**F**) Kaplan–Meier survival curves comparing the different treatment groups. PrimeC combination-treated ALS iMNs were compared to vehicle-treated ALS iMNs or ALS iMNs treated with monotherapies. *** *p* < 0.00025.

The survival of ALS patient-derived iMNs was assessed following treatment with the PrimeC combination, each individual compound, and control conditions. Longitudinal analyses were conducted to compare cell survival rates across all treatment groups (Figure 1A–C).

Initial longitudinal viability analysis over the entire 16-day observation period revealed a substantial drop in cell survival between days 14 and 16 across all treatment conditions and controls, including ALS and healthy iMNs. This decline was likely attributable to senescence-related degeneration, independent of treatment effects, a common occurrence under prolonged in vitro culture conditions. Therefore, to ensure accurate comparisons, data from day 16 were excluded from survival analyses, and all comparisons were conducted over a standardized 14-day period.

As expected, comparison of untreated control conditions demonstrated significantly higher survival rates in healthy control iMNs compared to ALS patient-derived iMNs treated with the vehicle (hazard ratio [HR] = 0.5, *p* < 0.001; Figure 1D–F). The median survival time of vehicle-treated healthy control iMNs was significantly longer than vehicle-treated ALS iMNs (12 days vs. 8 days, *p* < 0.001; Figure 1D), confirming the expected increased neurodegeneration characteristic of ALS-derived MNs.

Treatment of ALS iMNs with the PrimeC combination (ciprofloxacin + celecoxib) significantly improved neuronal survival compared to the vehicle control (HR = 0.46, *p* < 0.001). The median survival time of PrimeC combination-treated iMNs was extended compared to the vehicle control (12 days vs. 8 days; *p* < 0.001; Figure 1D–F).

Furthermore, the PrimeC combination demonstrated superior efficacy compared to monotherapy treatments. ALS iMNs treated with the PrimeC combination survived significantly longer than those treated with ciprofloxacin alone (HR = 0.63, *p* < 0.001), with a median survival time of 12 days for PrimeC combination-treated iPSCs versus 8 days for ciprofloxacin treatment alone (*p* < 0.001; Figure 1D–F). Similarly, PrimeC combination-treated ALS iMNs showed significantly improved survival compared to those treated with celecoxib alone (HR = 0.51, *p* < 0.001), extending median survival time for PrimeC combination-treated vs. celecoxib-treated ALS iMNs (12 days vs. 8 days; *p* < 0.001; Figure 1D–F).

Moreover, the survival of ALS iMNs treated with the PrimeC combination slightly exceeded that of the healthy control iMNs (HR = 0.84, *p* = 0.013), suggesting that the PrimeC combination may enhance neuronal resilience under the assay conditions (Figure 1D–F).

Collectively, these results demonstrate that the PrimeC combination provides a significant survival advantage in ALS patient-derived MNs compared to either monotherapy or untreated conditions, underscoring its potential therapeutic benefit.

### 2.2. Enhanced Brain Exposure Following PrimeC Combination Administration in Mice

**Figure 2:**
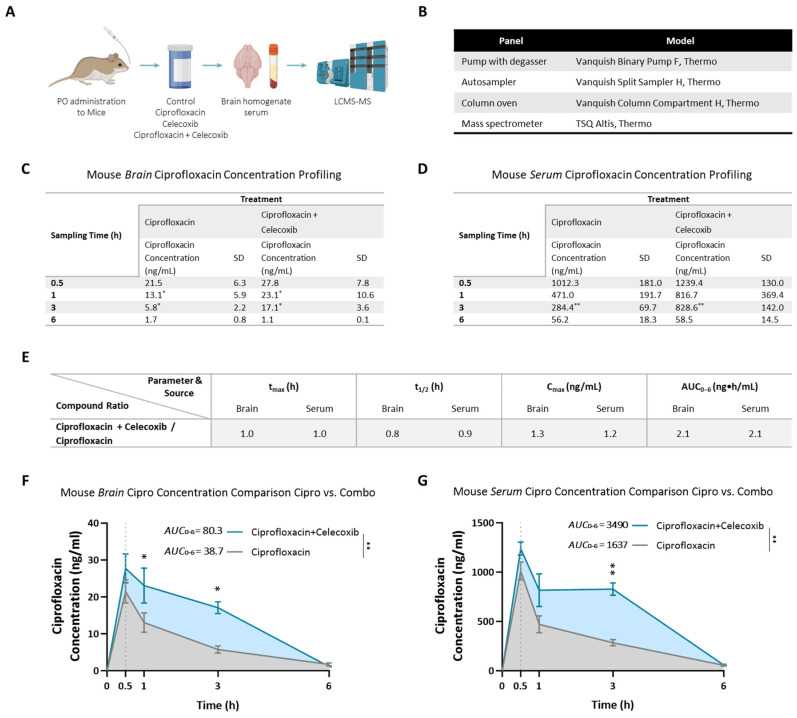
(**A**) Schematic overview of the mice drug concentration profiling study design. Fifty-eight male C57BL mice were divided into twelve treatment groups and administered a single oral solution dose of either ciprofloxacin alone or the PrimeC combination (ciprofloxacin + celecoxib). Each group was sacrificed at predetermined timepoints up to 6 h post-administration, followed by collection of blood serum and brain tissue samples for analysis. (**B**) LC-MS/MS system setup used for quantification of ciprofloxacin concentrations in brain and serum samples. System setup ensuring precise and consistent measurements. (**C**) Mean ciprofloxacin concentrations measured in brain samples at each time point following administration of either ciprofloxacin alone or the PrimeC combination. (**D**) Mean ciprofloxacin concentrations measured in serum samples following a single PO administration of ciprofloxacin alone or in combination with celecoxib. (**E**) Ratio of ciprofloxacin concentrations between the PrimeC combination-treated group and the ciprofloxacin-alone group across brain and serum samples at multiple time points. The ratios demonstrate consistently higher ciprofloxacin levels in the combination-treated group. (**F**,**G**) Mice drug concentration profiling study results. (**F**) AUC₀_–_₆ for brain samples, comparing ciprofloxacin exposure following PrimeC combination administration versus ciprofloxacin alone. (**G**) AUC₀_–_₆ for serum samples, comparing ciprofloxacin exposure following PrimeC combination administration versus ciprofloxacin alone. Gray-shaded areas represent ciprofloxacin monotherapy, while light-blue areas depict the combination treatment. Comparisons between treatment groups were evaluated using two-way ANOVA with Bonferroni post hoc test. * *p* ≤ 0.05, ** *p* < 0.01. The dotted line at 0.5 h marks the start of administration. The ascending lines from 0 h to this point are for visualization purposes only.

The drug concentration profile of the PrimeC combination was evaluated in C57BL mice following a single oral solution dose of either ciprofloxacin alone or the PrimeC combination (Figure 2A,B). Drug concentrations were measured in both brain tissue and serum to assess potential differences between treatment groups (Figure 2C,D).

Ciprofloxacin concentrations were detectable at all time points in both brain and serum samples across all treatment groups. In both groups, the time to reach maximum ciprofloxacin concentration (t_max_) was 0.5 h post-administration in both brain and serum. Ciprofloxacin levels declined in a log-linear manner starting at 0.5 h after dosing. The terminal half-life (t_1/2_) of ciprofloxacin was calculated as 1.6 h (brain) and 1.4 h (serum) in the ciprofloxacin-treated group, and 1.2 h (brain) and 1.3 h (serum) in the PrimeC combination-treated group.

Analysis of total drug exposure (AUC_0–6h_) showed a 108% increase in ciprofloxacin exposure in the brain following PrimeC combination treatment (AUC_0–6h_ = 80.27 ng·h/mL) compared to ciprofloxacin alone (AUC_0–6h_ = 38.69 ng·h/mL; 95% CI [−10.97, −2.563], *p* < 0.001; Figure 2E,F). Serum AUC_0–6h_ showed a 113% increase in the PrimeC group (AUC_0–6h_ = 3490 ng·h/mL) compared to ciprofloxacin alone (AUC_0–6h_ = 1637 ng·h/mL; 95% CI [−419.2, −140.5], *p* < 0.001; Figure 2E,G).

Notably, ciprofloxacin concentrations were consistently higher in the PrimeC combination group compared to ciprofloxacin alone. Peak ciprofloxacin concentration (C_max_) in brain tissue increased by 30% in the PrimeC combination-treated group (C_max_ = 27.8 ng/mL) compared to the ciprofloxacin-treated group (C_max_ = 21.5 ng/mL; Figure 2C,E,F). Serum C_max_ showed a 20% increase in the PrimeC combination-treated group (C_max_ = 1239.4 ng/mL) relative to the ciprofloxacin-treated group (C_max_ = 1012.3 ng/mL; Figure 2D,E,G).

Statistical analysis demonstrated significant differences in ciprofloxacin concentration profiles between the PrimeC combination and ciprofloxacin-alone groups throughout the study period, both in brain (F (1,8) = 11.72, *p* = 0.009; Figure 2F) and serum (F (1,8) = 18.74, *p* = 0.003; Figure 2G). Bonferroni post hoc analysis revealed significant differences favoring the PrimeC combination-treated group at 1 h (Mean Difference = 10.2, 95% CI [0.1127, 19.93], *p* = 0.047) and 3 h (Mean Difference = 11.34, 95% CI [1.428, 21.25], *p* = 0.020) in brain tissue (Figure 2C,E,F) and at 3 h in serum (Mean Difference = −544.2, 95% CI [−796.3, −292.1], *p* = 0.001; Figure 2D,E,G).

Overall, these results demonstrate that the PrimeC combination enhances ciprofloxacin exposure in both brain tissue and serum compared to ciprofloxacin administered alone. The increased concentrations observed with the PrimeC combination treatment suggest that the combination may improve CNS bioavailability.

## 3. Discussion

This paper examined the synergistic effect of ciprofloxacin and celecoxib in vitro as a combination treatment in an iPSC-derived MN model and evaluated the drug concentration profile of the PrimeC combination in vivo in mice. The safety, superior drug concentration profile, and synergistic neurotherapeutic effects of the PrimeC combination support its potential as a novel therapeutic agent for ALS.

Human iPSC-derived iMN models serve as a valuable platform for enabling drug screening approaches aimed at identifying compounds and combinations that can mitigate the impact of ALS pathophysiology. Peripheral blood mononuclear cells (PBMCs) are a type of white blood cells commonly used for generating iPSCs due to their ease of collection and reprogramming [20]. The lymphocytes from the human PBMCs are then reprogrammed into iPSCs. Cellular reprogramming, and particularly the generation of iPSCs, has significant benefits for drug development, as they can be generated from patient-specific cells and differentiated into various cell types. Deriving patient-specific iMNs recapitulates the neurodegeneration involved in ALS, allowing for the creation of ALS in vitro models, which ultimately provides a platform for drug screening and testing [21]. Moreover, cellular reprogramming enables high-throughput, personalized drug screening directly on patient-derived neurons, providing a powerful platform for evaluating potential therapies such as PrimeC.

For over a decade, iPSCs have been utilized as a valid and valuable disease modeling platform, enabling high-throughput drug screening to identify therapeutic applications for diseases such as ALS [22]. Multiple research groups have established various ALS iPSC models as powerful tools for recapitulating disease phenotypes manifested by neurodegeneration, cell death mechanisms, phenotypical progression, etc. [23]. For instance, ropinirole was identified as a potential therapeutic candidate for ALS in a large in vitro iPSC drug screening [23], which led to a Phase I/II trial [24,25]. In line with this approach, these iMNs derived from PALS recapitulate the neurodegeneration involved in ALS, thus providing a valid and biologically relevant in vitro model system [21,26].

In our experiments, PrimeC combination-treated ALS iMNs demonstrated survival rates comparable to those of healthy control iMNs and significantly higher survival compared to ALS iMNs treated with vehicle, ciprofloxacin alone, or celecoxib alone. ALS drug development has recently witnessed a spurt of iPSC-based studies, either for mechanism examination, safety, drug screening, or efficacy [23,24,27]. While these findings are promising, it is important to acknowledge that in vitro iPSC-derived models, despite their relevance, cannot fully recapitulate the complexity of ALS pathology [28].

The drug concentration profile study results demonstrated that ciprofloxacin exposure was higher following the administration of the PrimeC combination compared to ciprofloxacin alone. Overall, this proof-of-concept study in mice demonstrated that the combination of the two active substances improved the therapeutic mode of action relative to the administration of a single active drug. Previous studies have reported enhanced concentrations of ciprofloxacin in blood when administered in combination with other active substances targeting different pathologies [29]. However, this examination is the first of its kind in assessing the additive effect of ciprofloxacin with celecoxib, substantiating their combination as a viable therapeutic formulation.

These findings may suggest that the combination enhances ciprofloxacin’s retention in the brain. This observation aligns with prior studies showing that ciprofloxacin is a known substrate of multi-drug resistance 1 (MDR1) transporter [30], while celecoxib has been reported to inhibit MDR1, increasing intracellular ciprofloxacin levels [31]. Moreover, ALS-specific pharmacoresistance mechanisms have been identified, with selective upregulation of drug efflux transporters in the SOD1 G93A mouse model [32], reinforcing the relevance of MDR1 inhibition in ALS therapy. Additionally, COX inhibition has been associated with a 70–100% increase in cerebrospinal fluid (CSF) ciprofloxacin concentrations [33], supporting the hypothesis that celecoxib may enhance ciprofloxacin CNS retention. This potential for enhanced CNS exposure warrants further investigation, particularly in the context of treating neurodegenerative diseases.

Taken together, the results of this study demonstrate the beneficial and synergistic effects of ciprofloxacin and celecoxib as a combination therapy for ALS. This multifaceted approach addresses both improved drug concentration profiles and enhanced neuronal survival, substantiating the proof of concept for PrimeC. In conclusion, the aforementioned findings set the stage for the use of PrimeC as a potentially safe and effective treatment for ALS. Building upon these findings, future research should further explore PrimeC combination’s synergistic effects, incorporating mechanistic studies and human-equivalent models, advancing PrimeC toward better outcomes for PALS.

## 4. Materials and Methods

### 4.1. Induced Pluripotent Stem Cell (iPSC)-Derived Motor Neuron (MN) Survival

In this study, iPSC-derived iMNs were generated from PALS (C9orf72 pathology) and from healthy donors’ PBMCs. Neuronal survival rate was compared between PrimeC’s components, their combination, and the relevant DMSO vehicle reference as described in Figure 2A–C. Cell survival was measured by longitudinally tracking iMNs at days 2, 4, 6, 8, 10, 12, 14, and 16 post-treatment for each treatment condition.

#### 4.1.1. Peripheral Blood Mononuclear Cell (PBMC) Isolation

In this study, iPSC-derived iMNs were generated from PBMCs, which are a type of white blood cells commonly used for generating iPSCs due to their ease of collection and reprogramming [20]. Lymphoblastoid cell lines from healthy donors as well as from PALS were obtained from the National Institute of Neurological Disorders and Stroke (NINDS) Biorepository at the Coriell Institute for Medical Research. (C9 ALS: ND06769, ND10689, and ND12099; CTRLS: ND03231, ND03719, and ND05280).

#### 4.1.2. iPSC Generation

Lymphocytes sourced from individuals with ALS to generate iPSCs were then utilized. The lymphocytes were reprogrammed into iPSCs using episomal vectors—they were transfected with mammalian expression vectors carrying Oct4, Sox2, Klf4, L-Myc, Lin28, and a p53 shRNA using the Adult Dermal Fibroblast Nucleofector Kit and Nucleofector 2b Device (Lonza, Basel, Switzerland) following the manufacturer’s protocol. Subsequently, the cells were cultured on mouse feeders until iPSC colonies emerged. These colonies were then expanded and sustained on Matrigel (BD) in mTeSR1 medium (Stem Cell Technologies, Vancouver, BC, Canada) (following [34]).

#### 4.1.3. iPSC-Derived iMNs

iPSCs were differentiated into fibroblast-like cells to enable efficient retroviral transduction. Briefly, iPSCs were plated in several T-75 flasks coated with Matrigel, and the media was changed to fibroblast media (DMEM +10% FBS) at 30–50% confluency. The media was changed once a week until the appearance of fibroblast-like cells. The formation is cell line dependent and ranges from 30 to 50 days. Reprogramming experiments were conducted using 96-well plates. These were coated sequentially with gelatin (0.1% for 1 h at room temperature) and laminin overnight (4 °C, overnight). For each well of the 96-well plates, 7 iMN factors were added in 150 μL of fibroblast medium, along with 8 μg/mL of polybrene. Lentivirus encoding the Hb9::RFP reporter (labels MNs) was transduced into iMN cultures 48 h after the initial transduction with retroviruses encoding transcription factors. On day 4, primary mouse cortical glial cells derived from P2-P3 ICR pups (both male and female) were added to the transduced cultures in glia medium, which consisted of MEM (Life Technologies, Carlsbad, CA, USA), 10% donor equine serum (HyClone, Logan, UT, USA), 20% glucose (Sigma-Aldrich, St. Louis, MO, USA), and 1% penicillin/streptomycin. On day 5, the cultures were switched to N3 medium, containing DMEM/F12 (Life Technologies), 2% FBS, 1% penicillin/streptomycin, N2 and B27 supplements (Life Technologies), 7.5 μM RepSox (Selleck, Houston, TX, USA), and 10 ng/mL each of GDNF, BDNF, and CNTF (R&D). The iMN cultures were maintained in N3 medium, with medium changes performed every other day until day 16. Quality control was performed using Picard Tools AlignmentSummaryMetrics (following [34]).

#### 4.1.4. iMN Assay

Hb9:RFP+ iMNs form between days 13 and 16 after transduction of iMN factors. The iMN survival assay was initiated on day 17. Starting at Day 17, longitudinal tracking of iMNs was performed using Molecular Devices ImageExpress once every other day for 16 days. Tracking of neuronal survival was performed using ImageJ version 1.53t. Neurons were scored as dead when their soma was no longer detectable by RFP fluorescence. iMN survival experiments included neurotrophic factor withdrawal in order to exacerbate the survival difference between control and PALS’ iMNs. Neurotrophic factor withdrawal of FGF-2, BDNF, GDNF, and CNTF from the culture medium was performed on day 17. For treatment with drugs, cultures were treated with DMSO or a drug after neurotrophic factor withdrawal (starting at day 17). Treatment with fresh media containing vehicle or drugs occurred every 3 days for 16 days. In order to assess the different effects of PrimeC on the iPSC-derived C9orf72 cells, we used the following comparisons: iMNs treated with the combination dose of ciprofloxacin + celecoxib, cells treated with ciprofloxacin or celecoxib alone, and a vehicle control. Control cell lines of non-neurological subjects (i.e., neurologically healthy controls) were used in the comparisons. Three replicates per condition were used—three healthy control lines (*n* = 100 iMNs/line × 3 lines = 300 total iMNs) and three PALS lines (*n* = 100 iMNs/line × 3 lines = 300 total iMNs).

A hazard ratio was calculated for each of the comparisons as the likelihood of neuron death when treated with the indicated compound divided by the likelihood of neuron death when treated with another indicated compound. The lower the ratio, the more likely it is for a cell to die when treated with the second compound compared to being treated with the first compound in each comparison above, and vice versa.

#### 4.1.5. Statistical Analysis

Multiple groups and/or time points were analyzed utilizing two-way ANOVA, and when appropriate, a two-way ANOVA repeated measurements (time × groups).

Analysis utilized GraphPad Prism version 10.0.2 GraphPad. For iMN survival, a 2-sided log-rank test was employed to accommodate instances where events did not occur (iMNs that did not degenerate before the experiment’s conclusion). In each case, 300 iMNs were selected to generate survival curves. If all iMNs degenerated in a given experiment, a 2-tailed Student’s *t*-test was used for statistical significance.

The normal distribution of datasets was tested using the D’Agostino–Pearson omnibus normality test. Multiple group differences were assessed via one-way ANOVA with Tukey’s correction for all comparisons, unless the data were non-normally distributed, in which case nonparametric Kruskal–Wallis testing was applied. Normally distributed datasets use mean and standard deviation or standard error of the mean, while non-normally distributed datasets use median and interquartile range. Differences between two groups were analyzed using a 2-tailed Student’s *t*-test, unless the data were non-normally distributed, in which case 2-sided Mann–Whitney testing was utilized. Upon conducting Bonferroni correction for multiple comparisons, significance was assumed at *p* ≤ 0.0125.

Kaplan–Meier plots were generated using iMNs obtained from each treatment condition in replicates of three. Confirmation of iMN survival times was analyzed by manual longitudinal tracking.

### 4.2. Drug Concentration Profiling of the PrimeC Combination in C57BL Mice

The drug concentration profile of the PrimeC combination in C57BL strain mice’s brain and blood (serum) was tested following acute oral administration (PO; single dose). The drug concentration profile of ciprofloxacin alone was compared to the drug concentration profile of the combination compound of ciprofloxacin and celecoxib (i.e., the PrimeC combination). To this aim, a total of 58 male mice aged 8 weeks were utilized in this study, which was conducted in two cycles of 12 groups with 4–6 animals per group. The tested mice were PO administered (oral gavage) at time point 0 (see Figure 2A,B for study design).

#### 4.2.1. Mice

Given the variability in blood–brain barrier (BBB) pathology across different ALS mouse models [35], the use of a common mouse model, such as C57BL, allows for the assessment of drug effects on BBB permeability in the absence of model-specific pathological influences. This approach facilitates the identification of therapeutic effects with greater potential for broad applicability across diverse ALS phenotypes.

Hence, the C57BL strain male mouse model was used (Envigo RMS, Israel Ltd., Jerusalem, Israel). Animal handling was performed according to guidelines of the National Institute of Health (NIH) and the Association for Assessment and Accreditation of Laboratory Animal Care (AAALAC) and Pharmaseed standard operating procedures (SOPs). The animals were monitored for morbidity and mortality, body weight (BW), clinical signs, and bleeding during dosing times. This study was performed in compliance with “The Israel Animal Welfare Act” and following “The Israel Board for Animal Experiments” Ethics Committee # NPC-Ph-IL2109-110-3.

#### 4.2.2. Formulation Preparation

C57BL mice were treated with distinct concentrations of the PrimeC combination of celecoxib (Hikal Ltd., Karnataka, India) and ciprofloxacin (Neuland Laboratory Ltd., Telangana, India) or with ciprofloxacin alone.

The ciprofloxacin solution was dissolved in distilled water and stored at 2–8 °C. The celecoxib solution was dissolved in a mixture of DMSO and distilled water and was also stored at 2–8 °C. The solutions for both active pharmaceutical ingredients (APIs), ciprofloxacin and celecoxib, were prepared under appropriate conditions to yield specific predetermined concentrations. The solutions were administered via oral gavage in a sequential manner.

#### 4.2.3. Ciprofloxacin Liquid Chromatography with Triple Quadrupole Mass Spectrometer (LCMS-MS)

Blood and Serum Extraction

Approximately 350 μL of whole blood was collected into tubes containing a clotting activator gel. The blood was allowed to clot at room temperature for at least 30 min, followed by centrifugation at 4000 rpm for 10 min. The resulting serum was separated, transferred into Eppendorf tubes (or equivalent), and stored at −80 °C until further analysis for ciprofloxacin quantification using the LC-MS/MS method (see Figure 1B).

Brain Tissue Extraction

At the termination timepoints, animals were anesthetized via intraperitoneal injection of a ketamine–xylazine mixture (90:10 mg/kg, respectively) and briefly perfused with phosphate-buffered saline (PBS). Following sacrifice as approved by the Ethics Committee (Approval No. NPC-Ph-IL2109-110-3), brains were collected, weighed, and processed. Each brain was rinsed with ice-cold PBS, minced, and homogenized in 1 mL of ice-cold PBS using the gentleMACS™ Dissociator (Program Protein_01_01). The homogenates were then sonicated and centrifuged at 13,000× *g* for 10 min at 4 °C. The supernatant was aliquoted and stored at −80 °C until further analysis for ciprofloxacin quantification using the LC-MS/MS method (see Figure 1B).

LC-MS/MS Analysis

Both serum and brain supernatant samples were analyzed for ciprofloxacin quantification using the LC-MS/MS method.

Sample Preparation

Ciprofloxacin standard solutions were prepared by dissolving ciprofloxacin in methanol with 0.1% formic acid. Linearity standards, calibration standards, and quality control (QC) samples were prepared using serial dilutions in methanol with 0.1% formic acid. Blank mouse brain/serum extracts were spiked with these standards and an internal standard solution (Ciprofloxacin-d^8^), followed by vortexing (20 s) and centrifugation (15 min, 14,000 rpm, 4 °C). The resulting supernatant was transferred to vials for LC-MS/MS analysis.

LC-MS/MS Conditions

The system was equilibrated prior to sample injection, including system suitability test (SST) samples, blanks, calibrators, and QC samples. A calibration curve was constructed by plotting the peak area ratio of ciprofloxacin to the internal standard against nominal concentrations. System suitability criteria required %RSD ≤ 5% for consecutive injections of calibration standards, with retention time (RT) deviations within ±0.5 min of the expected RT. Data were acquired and processed using Chromeleon (v7.2.10 ES). The system met all acceptance criteria, ensuring reliable quantification of ciprofloxacin in both serum and brain supernatant samples.

#### 4.2.4. Pharmacokinetic Analysis

Pharmacokinetic parameters were calculated using the computer program PK Solutions 2.0 (Summit Research Services, Montrose, CO, USA). The following pharmacokinetic parameters were evaluated:

Maximum observed ciprofloxacin concentrations (C_max_) in serum and brain samples and their times of determination (t_max_) were the obtained values. Standard deviations were reported to the same precision as the corresponding mean value. Unless stated otherwise, all summary statistics (e.g., mean and SD) presented are based upon rounded numbers.

Areas under concentration–time curves up to the last quantifiable concentration (AUC_0–6_) were calculated using the linear trapezoidal rule. In the calculation of AUC_0–6_ values, it was assumed that the pre-dose (0 h) ciprofloxacin concentrations were zero. Data permitting, the terminal elimination rate constant (λZ) was estimated by fitting a linear regression of log concentration against time, and t_½_ was calculated as ln2/λZ. For the estimate of λZ to be accepted as reliable, the following criteria were imposed:

1. The terminal data points were apparently randomly distributed about a single straight line (on visual inspection).
2. A minimum of three data points was available for the regression.
3. The regression coefficients ≥ 0.85.
4. The interval including the data points chosen for the regression was at least two-fold greater than the half-life itself.

A mixed model for repeated measures followed by a post hoc Bonferroni test for multiple comparisons was performed for between-group comparisons of ciprofloxacin concentrations, with statistical significance set at *p* ≤ 0.05. This analysis was performed using GraphPad Prism version 10.0.2.

## 5. Conclusions

In conclusion, this integrated study, combining an in vitro iPSC-derived motor neuron survival assay and an in vivo brain and serum drug exposure study in mice, demonstrates the beneficial and synergistic effects of the PrimeC combination (ciprofloxacin and celecoxib) in addressing ALS-related pathology. The combination notably improves neuronal survival and enhances drug exposure profiles, supporting the proof of concept for PrimeC. This multidisciplinary approach substantiates the robust scientific rationale behind PrimeC, demonstrating its potential to enhance survival and improve drug concentration profiling. In summary, the aforementioned findings set the stage for the use of PrimeC as a potentially safe and effective treatment for ALS.

## Data Availability

Dataset is available upon request from the authors.

## Abbreviations

The following abbreviations are used in this manuscript:

ALS	Amyotrophic lateral sclerosis
AIDS	Acquired immunodeficiency syndrome
iPSCs	Induced pluripotent stem cells
PK	Pharmacokinetic
RNA	Ribonucleic acid
miRNA	MicroRNA
TRBP	Transactivation response element RNA-binding protein
RISC	RNA-induced Silencing Complex
NSAID	Non-steroidal anti-inflammatory drug
COX-2	Cyclooxygenase-2
CNS	Central nervous system
CSF	Cerebrospinal fluid
PALS	People living with ALS
TDP-43	TAR DNA-binding protein 43
PgJ2	15-deoxy-Δ12,14-prostaglandin J2
LC3	Microtubule-associated protein 1A/1B-light chain 3
ALSFRS-R	Revised Amyotrophic Lateral Sclerosis Functional Rating Scale
SVC	Slow vital capacity
FDA	Food and Drug Administration
MNs	Motor neurons
iMNs	Induced motor neurons
C9orf72	Chromosome 9 open reading frame 72
PBMCs	Peripheral blood mononuclear cells
DMSO	Dimethyl sulfoxide
NINDS	National Institute of Neurological Disorders and Stroke
C9	C9orf72 samples
CTRL	Controls
shRNA	Short hairpin RNA
Oct4	Octamer-binding transcription factor 4
Sox2	Sex-determining region Y-box 2
KLF4	Kruppel-like factor 4
L-Myc	L-myc-1 proto-oncogene protein
Lin28	Lin-28 homolog A protein gene
DMEM	Dulbecco’s modified eagle medium
FBS	Fetal bovine serum
MEM	Minimum essential medium
GDNF	Glial cell line-derived neurotrophic factor
BDNF	Brain-derived neurotrophic factor
CTNF	Ciliary neurotrophic factor
RFP	Red fluorescent protein
FGF-2	Fibroblast Growth Factor 2
ANOVA	Analysis of variance
PO	Per os
NIH	National Institute of Health
AAALAC	Association for Assessment and Accreditation of Laboratory Animal Care
SOP	Standard operating procedure
BW	Body weight
API	Active pharmaceutical ingredients
LCMS-MS	Liquid Chromatography with Triple Quadrupole Mass Spectrometer
mg	Milligram
kg	Kilogram
ng	Nanogram
mL	Milliliter
h	Hour
PBS	Phosphate-buffered saline
SD	Standard deviation
CI	Confidence interval
AUC	Area under the curve
t_1/2_	Terminal half life
t_max_	Transport maximum
C_max_	Maximum concentration
BBB	Blood–brain barrier
MDR1	Multi-drug resistance 1
